# A Case of Complete Epithelialization of an Extensive Foot Wound With Exposed Calcaneus Using Topical Cadexomer Iodine (CIOD) Monotherapy

**DOI:** 10.7759/cureus.95425

**Published:** 2025-10-26

**Authors:** George Miura, Norihiro Yoshimoto, Suguru Kurosawa, Yoichiro Oda, Shohei Hayashi, Hiroyuki Nakamura

**Affiliations:** 1 Dermatology, Kushiro City General Hospital, Kushiro, JPN; 2 Plastic Surgery, Kushiro City General Hospital, Kushiro, JPN

**Keywords:** bacaterial infection, cadexomer iodine, exudate, pressure ulcer, wound healing, debridement

## Abstract

Managing extensive chronic foot wounds with underlying bone exposure is challenging, particularly in patients who are poor candidates for extensive surgery. Cadexomer iodine (CIOD) is a hydrophilic, sustained‐release topical antimicrobial agent that has demonstrated efficacy in reducing bioburden, disrupting biofilms, and promoting autolytic debridement in chronic ulcers. To date, several reports have described epithelialization of small ulcers using CIOD alone, and others have documented epithelialization of larger ulcers when CIOD was combined with surgical procedures. However, no reports of complete epithelialization of large debrided wounds exposing bone with CIOD alone have been published. Herein, we report a case of complete epithelialization of a large debrided wound achieved with topical CIOD. This case highlights the potential utility of CIOD monotherapy for limb preservation through its sustained antimicrobial activity, inhibition of biofilm formation, control of exudate, and facilitation of autolytic debridement in high-risk patients.

## Introduction

Chronic foot wounds, particularly those with deep tissue or bone involvement, are associated with high morbidity and often require repeated surgical interventions or amputation [1]. Underlying factors such as poor perfusion, high bioburden, and microbial biofilms impede wound healing [2]. Biofilms are communities of microorganisms embedded in a self-produced extracellular polymeric substance (EPS). Biofilms are implicated in more than 78% of chronic, nonhealing ulcers and impede wound healing while compromising the effectiveness of antimicrobial agents [2,3].

Cadexomer iodine (CIOD) is a water-absorptive starch polymer bead infiltrated with iodine. When it contacts with exudate, it gels and continuously releases 0.9% iodine for up to 72 hours [1,2]. Clinical trials have demonstrated its efficacy in reducing bacterial load, including *Pseudomonas aeruginosa* and methicillin-resistant *Staphylococcus aureus* (MRSA), and in promoting autolytic debridement and exudate management in diabetic foot and venous leg ulcers [1,2,4,5]. In vitro and meta-analytic data support biofilm disruption [2].

Despite widespread use of CIOD in chronic ulcers and evidence of accelerated healing in relatively small or superficial wounds, to our knowledge, no reports describe complete epithelialization of a large, bone-exposed ulcer treated with CIOD solely following surgical debridement. In this report, we present a case of extensive calcaneal bone exposure that achieved complete wound closure with CIOD monotherapy following debridement, and we emphasize CIOD’s potential as a limb‐salvage strategy for patients with limited surgical options.

## Case presentation

**Figure 1:**
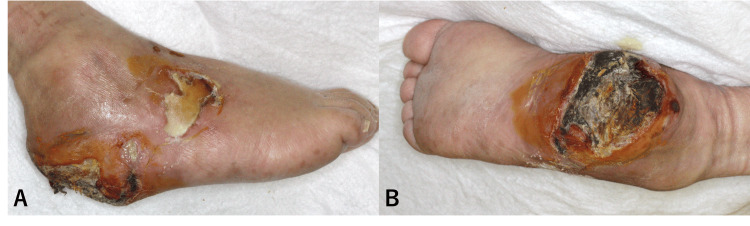
Condition of the right foot on admission A) Yellow necrotic tissue adhering in a map-like pattern on the dorsal and lateral aspects of the right foot. B) Nearly the entire right heel is covered with black necrotic tissue.

A 74-year-old man presented to our clinic with a severely painful, malodorous ulcer on the right heel that had developed approximately three months prior to admission. On examination, an 8 × 5 cm area of black necrosis was noted on the posterior heel, extending to yellow necrotic tissue over the lateral dorsum of the foot (Figure 1).

Laboratory investigations on admission (Table 1) demonstrated marked elevation of inflammatory markers. Wound cultures grew multiple enteric bacteria, including *Escherichia coli*. He was started on daily intravenous clindamycin and piperacillin-tazobactam.

**Table 1:** Laboratory investigations on admission

Parameter	Value	Reference range
White blood cell (×10³/µL)	27.9	3.3-8.6
Red blood cell (×10⁶/µL)	2.48	4.35-5.55
Hemoglobin (Hb) (g/dL)	7.6	13.7-16.8
Total protein (g/dL)	5.9	6.6-8.1
Albumin (g/dL)	2.0	3.8-5.2
Blood urea nitrogen (BUN) (mg/dL)	43.3	8-20
Creatinine (mg/dL)	1.56	0.65-1.07
Estimated glomerular filtration rate (mL/minute/1.73 m²)	34.8	≧60
Sodium (Na) (mEq/L)	136	138-145
Potassium (K) (mEq/L)	3.3	3.6-4.8
Alkaline phosphatase (U/L)	650	106-322
Aspartate aminotransferase (U/L)	58	13-30
Alanine aminotransferase (U/L)	57	10-42
Lactate dehydrogenase (U/L)	139	124-222
γ-glutamyltransferase (U/L)	108	13-64
Creatine kinase (U/L)	15	59-248
Hemoglobin A1c (NGSP) %	6.4	4.6-6.2
C-reactive protein (mg/dL)	28	＜0.3

Skin perfusion pressure (SPP) measured on the second hospital day was mildly decreased at 59 mmHg on the dorsal foot, 34 mmHg on the medial plantar surface, and 44 mmHg on the lateral plantar surface. The following day, the patient became hypotensive (systolic blood pressure in the 60s mmHg), and *Bacteroides fragilis* was isolated in two sets of blood cultures, consistent with septic shock. Given his severely reduced left ventricular ejection fraction and consultation with anesthesiology and cardiology, emergency surgical debridement was performed under local anesthesia with 1% lidocaine.

**Figure 2:**
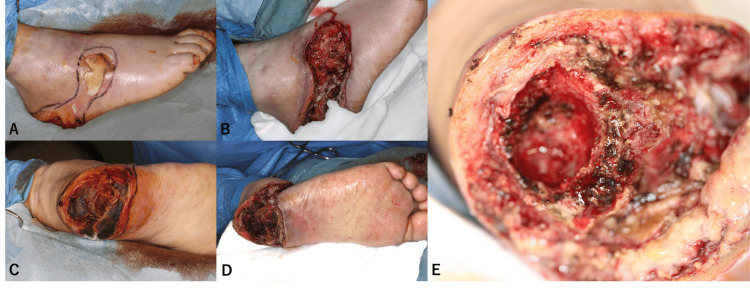
Preoperative and postoperative clinical photographs A) Preoperative view of the dorsal and lateral aspects of the right foot. B) Post-debridement. Almost all necrotic subcutaneous tissue is removed. C) Preoperative view of the right heel. D) Post-debridement. Necrotic tissue is present not only in the subcutaneous tissue but also in the calcaneus. E) A portion of the calcaneus is necrotic and is debrided.

Intraoperatively, full-thickness adipose tissue and necrotic bone, including a portion of the calcaneus, were excised, leaving exposed calcaneal cortex and tendon at the conclusion of the procedure (Figure 2).

Post-debridement, inflammatory markers declined sharply. Additional debridement, stump revision, or below-knee amputation were considered to be unable due to the patient’s poor cardiac reserve and high risk of recurrence, taking his immobility into account. A conservative approach using topical CIOD ointment alone was therefore adopted.

**Figure 3:**
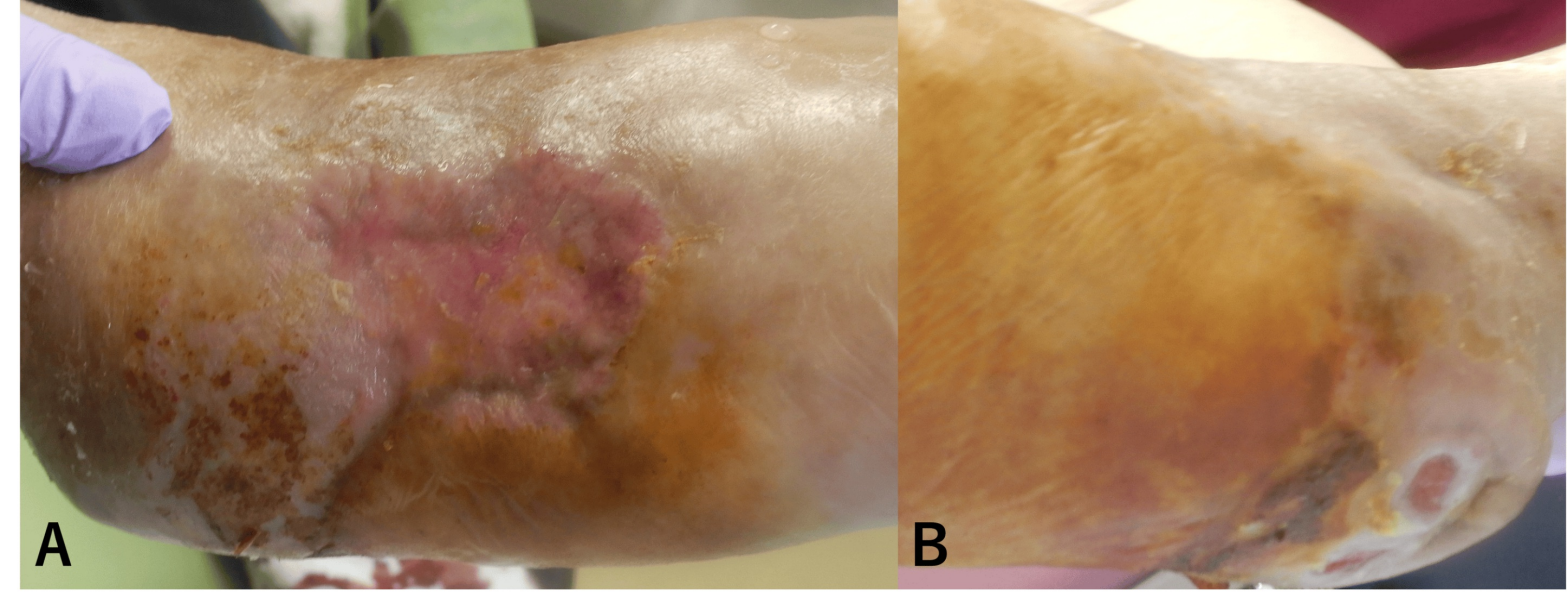
Clinical photographs eight months after surgery A) The dorsal foot has achieved complete epithelialization. B) The heel shows two small, round/oval erosions (black arrowheads), but epithelialization is nearly complete.

**Figure 4:**
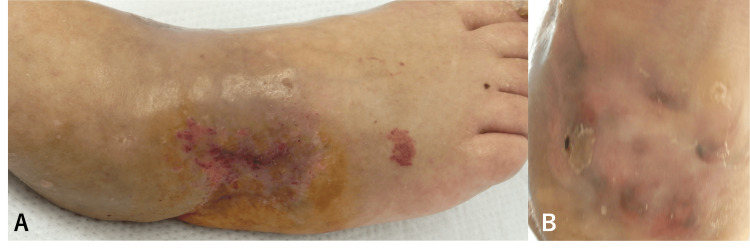
Clinical photographs one year after surgery A) The dorsal foot is completely epithelialized, with a few areas of purpura. B) The heel has also achieved complete epithelialization.

Despite the extensive debridement and bone exposure, progressive epithelialization was observed: near-complete coverage at eight months post-operatively (Figure 3) and full re-epithelialization by 10 months (Figure 4).

## Discussion

CIOD consists of hydrophilic cadexomer starch beads (100-315 μm in diameter) impregnated with iodine; upon absorbing wound exudate, the beads swell and gradually release 0.9% iodine, maintaining an effective iodine concentration in the wound and exerting sustained antimicrobial activity for up to 72 hours [1,2]. Consequently, CIOD is effective against bacteria such as *Pseudomonas aeruginosa* and MRSA on infected wound surfaces [1,4,5]. When CIOD contacts exudate, it forms a gel that facilitates autolytic debridement and slough removal, enhancing its utility in chronic wounds [2]. Furthermore, multiple in vitro models have demonstrated CIOD’s efficacy in disrupting microbial biofilms [2]. It is generally understood that biofilms - microbial communities embedded within a self-synthesized exopolymeric substance (EPS) - create dense architectures that protect bacteria from host antibodies, phagocytic cells, antibiotics, and disinfectants while enabling waste removal and nutrient uptake [3]. Woo et al. have shown that biofilms contribute to chronic wound infection and delayed healing, with over 78% of non-healing chronic ulcers containing biofilms [2]. A meta-analysis of topical antimicrobial therapies concluded that CIOD is the only agent shown in human clinical studies to reduce total microbial load in biofilm-laden wounds [6].

CIOD is indicated for pressure and skin ulcers, including burns and leg ulcers, and is widely used for chronic wounds such as diabetic foot ulcers and pressure ulcers. For example, Skog et al. reported high healing rates in infected chronic venous ulcers treated with CIOD [7], and Moberg et al. have demonstrated its wound-healing benefits in pressure ulcers [8]. Although many reports describe ulcer size reduction or epithelialization of relatively small wounds, to our knowledge, no published case documents complete epithelialization of a large, bone-exposed debrided wound treated solely with CIOD. Our outcome aligns with clinical and mechanistic evidence that CIOD lowers microbial burden and facilitates autolytic debridement in chronic wounds [2]. It expands the literature by documenting complete epithelialization in a large, debrided ulcer with bone exposure under CIOD monotherapy, an indication not previously reported to our knowledge. Wounds with heavy exudate and contamination are particularly amenable to CIOD’s combined exudate management and autolytic debridement properties. In a comparison with other dressings, a systematic review and meta-analysis published in 2024 provided robust evidence that silver dressings (e.g., silver sulfadiazine) significantly reduce time to wound healing compared with CIOD, while exerting no effect on overall healing rates [9]. However, the authors concluded that direct comparisons of topical agents are limited because of the inconsistency in assessment criteria, such as effects on exudate volume, pain, and antimicrobial efficacy in studies [9]. Notably, CIOD has been shown to have a 24-hour water-absorption capacity per unit weight 2.9 times greater than that of povidone-iodine, another iodine dressing [10]. This high absorptive capacity is widely exploited in clinical practice. Consequently, CIOD may be particularly well suited for wounds with heavy exudation. The success of CIOD therapy in the present case can be attributed to the large amount of exudate at the wound and the patient’s relatively preserved peripheral perfusion and nutritional status [11]. In patients for whom general anesthesia or amputation is not feasible, conservative therapy with CIOD may represent a highly effective limb-salvage option.

## Conclusions

We successfully achieved complete epithelialization of an extensive foot defect involving exposed calcaneal bone through CIOD monotherapy following surgical debridement. CIOD provides sustained antimicrobial activity, disrupts biofilms, controls exudate, and promotes autolytic debridement. These properties make it an effective topical therapy for chronic ulcers and a useful option when surgical intervention is not feasible. It can be safely applied at the bedside without specialized skills, supporting its use in resource-limited settings. We hope that additional cases will be reported, enabling clearer guidance on patient selection and practical use of CIOD monotherapy.
